# Supplementary material for: Inflammatory factors and risk of lung adenocarcinoma: a Mendelian randomization study mediated by blood metabolites
Source: Front Endocrinol (Lausanne). 2024 Aug 27;15:1446863. doi: 10.3389/fendo.2024.1446863 (PMC11384989; doi:10.3389/fendo.2024.1446863)
Supplement: Supplementary file 1 [file DataSheet1.zip › supplementary files/S2/GCST90200736/sensitivity-analysis.pdf]

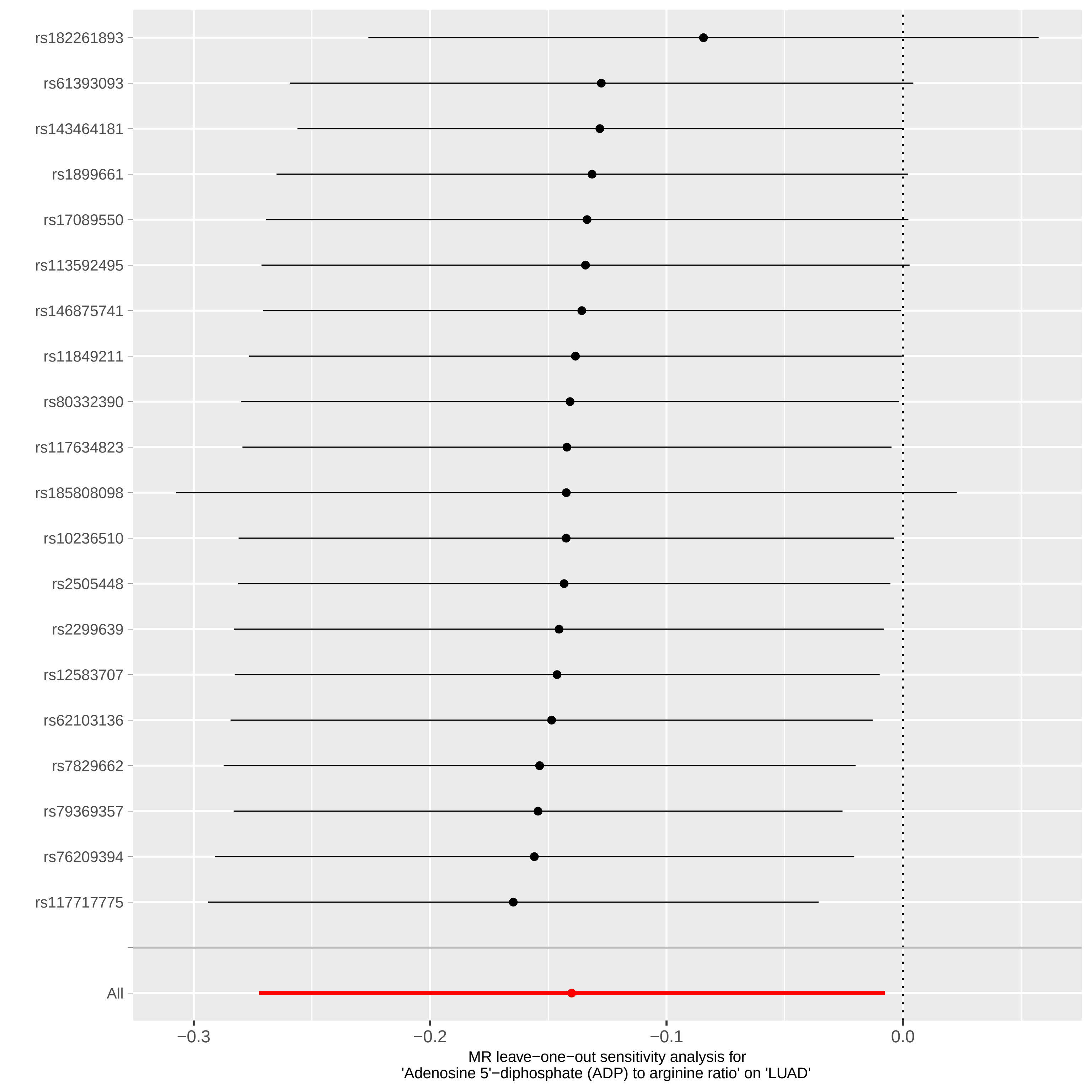
rs182261893
rs61393093
rs143464181
rs1899661
rs17089550
rs113592495
rs146875741
rs11849211
rs80332390
rs117634823
rs185808098
rs10236510
rs2505448
rs2299639
rs12583707
rs62103136
rs7829662
rs79369357
rs76209394
rs117717775
All
−0.3
−0.2
−0.1
0.0
MR leave−one−out sensitivity analysis for
'Adenosine 5'−diphosphate (ADP) to arginine ratio' on 'LUAD'
